# A Few Words in Reference to Some Medical Uses of Formaldehyde

**Published:** 1899-04

**Authors:** W. H. Hudson

**Affiliations:** Lafayette, Ala.


### A FEW WORDS IN REFERENCE TO SOME MEDICAL USES OF FORMALDEHYD.

BY W. H. HUDSON, M.D.,
LAFAYETTE, ALA.

I do not know that the facts in this paper have not been observed before, and therefore leave out the question of originality. Two summers ago I was consulted by a gentleman in reference to his feet. For years his feet had sweated, summer and winter, to such an extent that they had grown to be very troublesome. The odor from his feet was also very disagreeable, and though a man of cleanly habits, he found it impossible to destroy the odor either by frequent bathing or the use of antiseptic powders, such as boric acid, etc. His toes became very tender on account of the macerating effect of the constant moisture, and soft corns grew luxuriantly between several of his toes.

Some time before I had been consulted by this man I had been using a solution of formaldehyd as a hardening agent for pathological specimens, and once in injecting a specimen I had gotten my hands into the solution, and I observed that for several days afterward my hands were very *dry*, and that they appeared to become abnormally hard. With this fact in mind, I made an application of a solution of the formaldehyd, of about 50 per cent. strength. This caused the sweating to stop immediately and prevented the growth of the epithelium about the toes. When applied to the spots from which the corns were removed and the exuberant epithelial growth of the skin, around and between the toes, it produced a whitening of the tissues, and in a few days these points were perfectly smooth. In this case it was weeks that the feet did not sweat, and there could be no odor discovered even after days when the feet had not been bathed. This man finds that he can keep his feet in good condition by bathing them two or three times a month in a pan of water to which has been added a few teaspoonfuls of formaldehyd.

Later I have found that a 50 per cent. solution of the formaldehyd is by far too strong for use in sweating and odorous feet, although such a solution may be applied to corns, restricting the spreading of the solution so as not to harm the normal skin around the corn. For sweating and odorous feet, a few teaspoonfuls of the commercial formaldehyd may be added to a pan of hot water, or a 10 per cent. solution may be applied to the toes and soles of the feet with a swab of cotton held in a pair of forceps.

A strong solution, even the full strength of the formaldehyd, may be applied to ulcerating sarcomatous, or carcinomatous growths with great benefit. Indeed, there seems to be a field of usefulness for formaldehyd in any place where it would be of benefit to kill bacteria and chemical processes dependent on bacteria. Equally useful it is in such cases where it is desirous of killing cells and abnormal cell growth, as in inoperable ulcerating growths of any kind. It would, perhaps, in some cases, be possible to destroy such growths by the repeated application of the formaldehyd—removing the hardened dead portion some days after the application, and then applying again the formaldehyd, and so on until healthy tissues are reached.

---

### ORTHOFORM IN TOOTHACHE.

Dr. Hildebrandt (*Medical Press and Circular*) asserts that orthoform causes to cease completely the violent pain due to inflammation of the pulp of a decayed tooth. To this end it is sufficient to introduce into the cavity of the tooth a plug of cotton steeped in an alcoholic solution of orthoform. The pain instantly disappears, and for a considerable time. Being absolutely deprived of any toxic properties, orthoform constitutes in such cases a simple remedy, and one which the patient can apply himself without danger.

